# The CRISPR/Cas9-Mediated Modulation of *SQUAMOSA PROMOTER-BINDING PROTEIN-LIKE 8* in Alfalfa Leads to Distinct Phenotypic Outcomes

**DOI:** 10.3389/fpls.2021.774146

**Published:** 2022-01-05

**Authors:** Stacy D. Singer, Kimberley Burton Hughes, Udaya Subedi, Gaganpreet Kaur Dhariwal, Kazi Kader, Surya Acharya, Guanqun Chen, Abdelali Hannoufa

**Affiliations:** ^1^Lethbridge Research and Development Center, Agriculture and Agri-Food Canada, Lethbridge, AB, Canada; ^2^Department of Agricultural, Food and Nutritional Sciences, University of Alberta, Edmonton, AB, Canada; ^3^London Research and Development Center, Agriculture and Agri-Food Canada, London, ON, Canada

**Keywords:** crop improvement, drought tolerance, forage, genome editing, *Medicago sativa*, precision breeding

## Abstract

Alfalfa (*Medicago sativa* L.) is the most widely grown perennial leguminous forage and is an essential component of the livestock industry. Previously, the RNAi-mediated down-regulation of alfalfa *SQUAMOSA PROMOTER-BINDING PROTEIN-LIKE 8* (*MsSPL8*) was found to lead to increased branching, regrowth and biomass, as well as enhanced drought tolerance. In this study, we aimed to further characterize the function of *MsSPL8* in alfalfa using CRISPR/Cas9-induced mutations in this gene. We successfully generated alfalfa genotypes with small insertions/deletions (indels) at the target site in up to three of four *MsSPL8* alleles in the first generation. The efficiency of editing appeared to be tightly linked to the particular gRNA used. The resulting genotypes displayed consistent morphological alterations, even with the presence of up to two wild-type *MsSPL8* alleles, including reduced leaf size and early flowering. Other phenotypic effects appeared to be dependent upon mutational dosage, with those plants with the highest number of mutated *MsSPL8* alleles also exhibiting significant decreases in internode length, plant height, shoot and root biomass, and root length. Furthermore, *MsSPL8* mutants displayed improvements in their ability to withstand water-deficit compared to empty vector control genotypes. Taken together, our findings suggest that allelic mutational dosage can elicit phenotypic gradients in alfalfa, and discrepancies may exist in terms of *MsSPL8* function between alfalfa genotypes, growth conditions, or specific alleles. In addition, our results provide the foundation for further research exploring drought tolerance mechanisms in a forage crop.

## Introduction

The livestock industry depends critically on our ability to grow forage crops in a highly productive manner. Of the perennial leguminous forages, alfalfa (*Medicago sativa* L.) is the most widely grown, with an estimated global cropping area of approximately 30 million hectares (Singer et al., 2018a,b). The prominence of this crop derives from its many beneficial characteristics, including its perennial nature, relatively high yield, quality and palatability (Radović et al., 2009). Alfalfa’s symbiotic relationship with *Rhizobium* spp., which allows for nitrogen fixation, also enhances soil nutrition and reduces fertilization needs. Since the global demand for livestock-derived products is predicted to escalate in line with population growth and rising affluence, and the land base for forage production is decreasing (Kingston-Smith et al., 2013), there is a vital need for the provision of new alfalfa cultivars with superior productivity in the face of future climate change scenarios in a relatively short timeframe (Singer et al., 2018a). While conventional breeding approaches have been used to enhance various traits in alfalfa (Smith et al., 2000; Acharya and Steppuhn, 2012; Acharya, 2013), it can be challenging in this species due to its typically allogamous reproductive behavior, high levels of genetic variation and environmental interactions. Therefore, the time required for cultivar development using these approaches can be prohibitive (Annicchiarico et al., 2015) and thus achieving expeditious improvements in certain traits will undoubtedly necessitate the use of modern biotechnological approaches as a complementary platform in order to meet future demand.

SQUAMOSA PROMOTER-BINDING PROTEIN-LIKE (SPL) proteins are a family of plant-specific transcription factors that possess a highly conserved zinc-containing DNA binding region termed the SBP-domain (Yamasaki et al., 2004; Birkenbihl et al., 2005) and function in a partially overlapping manner in the regulation of an exceptionally diverse set of processes such as vegetative growth, response to abiotic stress and yield (Yamasaki et al., 2009; Xing et al., 2013; Xu et al., 2016; Gao et al., 2018a; Hu et al., 2021). Many *SPL* genes are targeted by microRNA156 (miR156), which is an abundant and highly conserved miRNA in plants that regulates the expression of its target genes through transcript cleavage and translational inhibition (Gandikota et al., 2007; Gao et al., 2016). While this group of transcription factors tends to be well-conserved across monocot and eudicot plant species (Wang et al., 2009), family members may have distinct functions between species in certain cases (Preston et al., 2016; Gou et al., 2019; Cao et al., 2021).

In alfalfa, a total of sixteen *SPL* genes have been identified to date, ten of which are directly silenced by miR156 (Gao et al., 2016; Feyissa et al., 2021). Intriguingly, while *SPL8* does not appear to be a target of miR156 in *Arabidopsis thaliana* (Xing et al., 2010), it is cleaved by miR156 in alfalfa (Feyissa et al., 2021). This, along with the fact that there is considerable functional disparity among *SPL8* homologs in different plant species (Unte et al., 2003; Zhang et al., 2007; Xing et al., 2013; Gou et al., 2018, 2019), suggests that it may have evolved in a distinct manner at some point following the divergence of particular lineages. In alfalfa, plants with RNAi-mediated down-regulation of *MsSPL8* have been found to exhibit many similarities to miR156 over-expression genotypes, including increased forage biomass production through enhanced branching and accelerated regrowth, as well as superior feed value and tolerance to drought and salinity (Gou et al., 2018). Unfortunately, despite the potential of such genotypes for economic benefit, their implementation will almost certainly be hindered by their transgenic nature. Since crops developed using RNAi technology are considered to be “genetically modified” (“GM”), they would raise public concern and be subject to prohibitively costly and lengthy regulatory processes (Singer et al., 2021a).

Genome editing technology based on CRISPR/Cas (Jinek et al., 2012) is a relatively new breeding platform that in its simplest form involves the endogenous non-homologous end-joining (NHEJ)-mediated repair pathway, which typically leads to the directed production of a small insertion or deletion (indel) at the selected target site. This platform requires a Cas nuclease, which produces a double-stranded DNA break, and a single guide RNA (sgRNA), which includes a short user-defined sequence that guides the Cas nuclease to a highly specific chromosomal locus of choice immediately upstream of a protospacer-adjacent motif (PAM). Due to the unlinked nature of the resulting edit and the introduced transgene, this technology allows for the rapid production of non-transgenic germplasm bearing mutations that are identical in nature to those achieved spontaneously or through conventional breeding approaches such as chemical mutagenesis (Subedi et al., 2020a,b). While the regulatory status of crop varieties derived from genome editing is still uncertain in some countries, many others, including the United States, have concluded that in the absence of foreign DNA they are not “GM,” and will therefore not be subjected to costly and burdensome regulatory processes (Schmidt et al., 2020; Singer et al., 2021a).

CRISPR/Cas has proven to be very effective in a range of polyploid plant species (Morineau et al., 2017; Liu et al., 2018; Huang et al., 2020), including alfalfa (Chen et al., 2020; Wolabu et al., 2020), due to its ability to simultaneously mutate multiple alleles of a target gene in a single generation. As such, our aim was to assess the effectiveness of CRISPR/Cas9 technology in alfalfa in terms of eliciting phenotypic alterations along a scale of mutational dosages by targeting the *MsSPL8* gene, and also to gain a further understanding of the functionality of this gene in this species. In addition, the successful exploitation of this technology in this context could potentially allow for the downstream generation of transgene-free germplasm exhibiting improvements in traits such as adaptability to water-deficit, which would provide a novel source of germplasm for alfalfa breeding endeavors in the future.

## Materials and Methods

### Plant Material and Growth Conditions

The highly regenerable N4.4.2 alfalfa genotype (Badhan et al., 2014) was utilized for transformation experiments throughout this study, while the cultivar AC Blue J (provided by Dr. Surya Acharya, AAFC Lethbridge) was used for crossing. Plants were grown in square pots (10.5 cm across and 12.5 cm in height) filled with Cornell soilless potting mix (Boodley and Sheldrake, 1972) in a greenhouse with supplemental lighting providing a 16 h/8 h photoperiod, a temperature of approximately 20°C/15°C day/night, and a light intensity of approximately 150 μmol/m^2^/s. Unless otherwise specified, plants were watered daily (without fertilizer) from the bottom for 15 min, after which time any excess water was drained off. Due to alfalfa’s obligatory outcrossing nature, the N4.4.2 background genotype, as well as all transgenic plants generated in this study, were propagated vegetatively through rooted stem cuttings to preserve particular genotypes throughout the experiment and generate “pools” of individual genotypes for downstream investigations. In every case, rooted cuttings were transferred to pots at the same time point, and were re-grown after cutting back to approximately 5 cm at least twice prior to assessments. During all trials, pots were rotated randomly every other day to minimize variations due to microclimate effects.

### Identification of *MsSPL8* Homologs and Expression Analysis

BLAST searches were performed against the *M. sativa* cultivated alfalfa at the diploid level (CADL; v0.95 and v1.0) genome database^1,2^, the Alfalfa Gene Index and Expression Atlas database^3^ and the tetraploid alfalfa genome sequence (Chen et al., 2020; NCBI database project PRJNA540215) using the *Medicago truncatula* Medtr8g005960 sequence, which encodes the *SPL8* homolog in this species (Gou et al., 2018). Nucleotide and amino acid alignments were carried out on identified homologs using Geneious Prime software (Biomatters Inc., San Diego, CA, United States), while domain searches were conducted using SMART^4^. Expression of alfalfa *MsSPL8* homologs was screened using the gene expression profiling tool in LegumeIP V3 (Dai et al., 2021) for *M. sativa* (CADL; v1.0) in different tissues.

### Generation of Binary CRISPR Vectors

Three separate guide RNAs (gRNAs; 20-nt) were designed immediately upstream of 5′—NGG—3′ PAM sequences (Doench et al., 2014) within a conserved region of the first exon of the four highly similar *MsSPL8* alleles (chr 8.2 83866861:83868757, chr 8.3 81917086:81918958; chr 8.4 80839466:80841286 and 80871132:80872948; Figure 1A). All three gRNAs exhibited 100% complementarity with all four *MsSPL8* alleles (as did all primers and probes utilized in this study). The specificity and/or potential for off-target effects of each gRNA was evaluated using the Cas-OFFinder algorithm (Bae et al., 2014). Each gRNA was inserted into the binary pKSE401 vector (Xing et al., 2014; Addgene plasmid #62202). This background vector includes a cassette in which the expression of the sgRNA is driven by the Arabidopsis U626 polymerase III promoter and another in which the expression of a *Zea mays* codon-optimized *Cas9* coding region (derived from *Streptococcus pyogenes* and flanked on either side by nuclear localization signals) is driven by a constitutive partially duplicated CaMV 35S promoter. This vector is designed to elicit NHEJ-mediated edits within the target gene.

**FIGURE 1:**
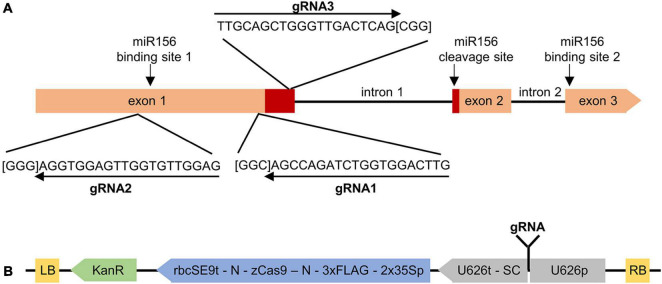
Targeting *MsSPL8* alleles for NHEJ-based editing using CRISPR/Cas9. **(A)** Genomic structure of *MsSPL8* alleles and gRNA target regions. The region encoding the SBP domain is displayed in red and PAM sequences are indicated in square brackets. miR156 binding and cleavage sites are indicated with arrows. **(B)** Three gRNAs targeting different regions of *MsSPL8* alleles were inserted into the pKSE401 background vector to yield SPL8-gRNA1, SPL8-gRNA2 and SPL8-gRNA3 binary vectors. 2x35Sp, partially duplicated CaMV 35S promoter; 3xFLAG, polypeptide tag; KanR, kanamycin resistance cassette; LB, left border; N, nuclear localization signal; RB, right border; rbcSE9t, pea RuBisCO small subunit E9 terminator; SC, sgRNA scaffold; U626p; Arabidopsis U6 polymerase III promoter; U626t; U6 terminator; zCas9, *Zea mays* codon-optimized Cas9.

In each case, 20-nt oligonucleotides corresponding to each gRNA in both orientations were designed and then synthesized by a third-party [Integrated DNA Technologies (IDT) Inc., Coralville, IA, United States]. Each oligonucleotide possessed 4-nt additions engineered at their 5′ termini to facilitate cloning into a *Bsa*I site within pKSE401 (5′—ATTG—3′ for forward oligonucleotides and 5′—AAAC—3′ for reverse oligonucleotides; Xing et al., 2014). Equimolar ratios of each oligonucleotide pair were then annealed by incubating at 65°C for 5 min, followed by cooling at room temperature. Each double-stranded gRNA was then ligated into pKSE401 that had been linearized with *Bsa*I (Figure 1B). All three of the resulting vectors were subjected to sequencing to confirm their identities.

### Alfalfa Transformation

The three CRISPR/Cas9 binary vectors targeting three distinct regions of *MsSPL8* alleles, as well as the empty vector (pKSE401), were introduced into *Agrobacterium tumefaciens* LBA4404 using electroporation and transferred separately into the alfalfa N4.4.2 genotype through *Agrobacterium*-mediated transformation of leaf explants as described previously (Badhan et al., 2014). Selection was carried out using 50 mg/L kanamycin for 10 days followed by 75 mg/L kanamycin for the remainder of tissue culture. Kanamycin-resistant genotypes were transferred to water in 15 mL polypropylene tubes for 3–4 days for acclimation, and subsequently transplanted to pots under greenhouse conditions with supplemental light. To confirm the presence of the transgenic cassette in kanamycin-resistant genotypes, genomic DNA was extracted from leaf tissues using the BioSprint 96 DNA Plant Kit (Qiagen Inc., Toronto, ON, United States) and the presence of each transgene was verified by PCR using Invitrogen Platinum SuperFi Green PCR Master Mix (Thermo Fisher Scientific, Waltham, MA, United States) and primers Cas9F1 and Cas9R1 (see Supplementary Table 1 for primer sequences; 621-bp amplicon). These primers anneal to the *Cas9* coding region within the pKSE401 transgenic cassette, and allow the differentiation of genotypes based on the presence/absence of the transgene. Thermal parameters included an initial denaturation step of 98°C for 30 s, 35 cycles of 98°C for 10 s, 57°C for 10 s, and 72°C for 30 s, and a final extension step of 72°C for 1 min. Positive control PCRs were conducted using GoTaq Green Master Mix (Promega Corp., Madison, WI, United States) and primers WD40F2 and WD40R2 (see Supplementary Table 1 for primer sequences; 865-bp amplicon) to ensure DNA quality. Thermal parameters included an initial denaturation step of 95°C for 2 min, 35 cycles of 95°C for 30 s, 51°C for 30 s and 72°C for 1 min, and a final extension of 72°C for 5 min. Primary transformants were labeled SPL8-gRNA1, SPL8-gRNA2 or SPL8-gRNA3 according to the gRNA used, while genotypes bearing the pKSE401 empty vector were termed EV.

### Droplet Digital PCR

Gene editing frequencies (GEF) were assessed using GEF-droplet digital PCR (GEF-ddPCR; Mock et al., 2016), which used two distinct probes that anneal within a single amplicon to quantify wild-type and NHEJ-affected alleles. Primers were designed using the PrimerQuest Tool (IDT). Primers MsSPL8Fwd2 and MsSPL8Rev2 were used to amplify a 375-bp region surrounding the potential gRNA1 and gRNA2 editing sites, while primers MsSPL8Fwd3 and MsSPL8Rev3 were used to amplify a 122-bp region surrounding the potential gRNA3 editing site (see Supplementary Table 1 for primer sequences). Locked nucleic acid (LNA) probes (Tm = 66°C, 5′ hexachloro-fluorescein [HEX] labeled) were designed to bind to predicted Cas9 cut sites in each of the three gRNA target regions as a means of detecting indels. Control probes (Tm = 68°C, 5′ fluorescein amidite [FAM] labeled) were also designed to bind to a region within each amplicon that would not be impacted by gene editing (see Supplementary Table 2 for probe sequences). All probes bore a 3′ Iowa Black fluorescent quencher. Mini-genes representing the gRNA3 target region with a single nucleotide insertion (123-bp in length) or deletion (121-bp in length) were synthesized (IDT) and utilized as positive controls to validate the sensitivity and specificity of the method in alfalfa.

Genomic DNA from leaves derived from a single shoot of all confirmed independent T_0_ transgenic genotypes, as well as five independent transgenic EV genotypes, was used as template for the initial screening of all three gRNA target sites. Subsequently, genomic DNA from leaves derived from eight separate and randomly chosen primary branches of three independent T_0_ SPL8-gRNA1 genotypes (SPL8-gRNA1-1, -2 and -3), as well as an EV control, respectively, was assayed as a means of examining the possibility of mosaicism in genotypes bearing a mutation at the gRNA1 cleavage site.

Assays were performed using ddPCR Supermix for Probes (no dUTP; Bio-Rad Laboratories Ltd., Mississauga, ON, Canada) following the manufacturer’s instructions. Briefly, 20 μL reactions containing 900 nM of each primer, 250 nM of the appropriate probe and 40 ng of genomic DNA were utilized for assays, and droplets were generated using the Automated Droplet Generator (Bio-Rad). Thermal cycling was performed using a C1000 Touch Thermal Cycler with 96-deep well reaction module (Bio-Rad) with parameters of 95°C for 10 min, 40 cycles of 94°C for 30 s and 60°C for 1 min, followed by enzyme deactivation at 98°C for 10 min. Droplets were then analyzed with the QX200 Droplet Reader (Bio-Rad). Analysis thresholds were established for each assay and the ratio of NHEJ-sensitive vs. NHEJ-insensitive droplets, which is based on the concentrations of events per μL, was calculated using the QuantaSoft Analysis Pro software V 1.0.596 (Bio-Rad). To quantify the relative number of edited *MsSPL8* alleles compared to total alleles within each sample, GEF was calculated as the percentage of edited droplets. In the case of T_0_ SPL8-gRNA1 genotypes, all subsequent experiments were carried out using plants derived from vegetative stem cuttings of the shoot exhibiting the highest level of GEF in the original T_0_ genotype.

### T7E1 Assay

To provide further confirmation of editing in SPL8-gRNA1 genotypes, T7E1 mismatch cleavage assays were also carried out using genomic DNA isolated from SPL8-gRNA1-1, -2 and -3 genotypes (with GEF of approximately 75, 50, and 25%, respectively), as well as three independent EV controls, as template. Primers MsSPL8F2 and MsSPL8Rev2 were designed to amplify a 255-bp region of *MsSPL8* encompassing the predicted editing location for gRNA1 (see Supplementary Table 1 for primer sequences). In the presence of an indel at the predicted cut site, and hence cleavage of the heteroduplex by the T7E1 enzyme, fragments of 156-bp and 99-bp in length would be generated. PCR amplification was carried out using Invitrogen Platinum SuperFi Green PCR Master Mix (Thermo Fisher Scientific) and a thermal program of 98°C for 30 s, 35 cycles of 98°C for 10 s, 55°C for 10 s and 72°C for 30 s, and a final extension at 72° for 5 min. Heteroduplexes of each PCR amplicon were formed by denaturing for 10 min at 95°C, cooling to 85°C at a ramp rate of 2°C/s, and then cooling to 25°C at a ramp rate of 0.3°C/s before digestion with 10 U of T7EI (New England Biolabs, Whitby, ON, Canada) at 37°C for 15 min. Digested products were separated and visualized on a 2% agarose gel.

### Assessment of On- and Off-Target Editing Events in SPL8-gRNA1 Genotypes

T_0_ transgenic SPL8-gRNA1-1, -2 and -3 genotypes (exhibiting GEF of approximately 75, 50, and 25%, respectively) were examined to confirm on-target mutations, and assessed for possible off-target mutations, using Sanger sequencing. To determine precise editing events at the gRNA1 target site, a 1.4-kb region of *MsSPL8* was amplified from genomic DNA derived from each genotype (including EV controls) using Invitrogen Platinum SuperFi Green PCR Master Mix (Thermo Fisher Scientific) and primers MsSPL8F1 and MsSPL8R2 (see Supplementary Table 1 for primer sequences). The amplicons were subsequently cloned into the pGEM-T-Easy vector (Promega), and clones from 21–33 colonies were subjected to Sanger sequencing (Génome Québec Centre D’Expertise et de Service, Montreal, QC, Canada).

In addition, the three most likely potential off-target sites were also chosen for evaluation, possessing two single nucleotide mismatches and a 2-nt RNA bulge (two sites), or three single nucleotide mismatches and a 1-nt RNA bulge (one site; Supplementary Figure 1) based on findings obtained using the Cas-Offinder program. In each case, an approximately 700-bp region spanning the potential off-target site was amplified from the same three transgenic SPL8-gRNA1 genotypes, as well as untransformed N4.4.2 and an EV genotype using primers MsOffTarget1FWD1 and MsOffTtarget1REV1, MsOffTtarget2FWD1 and MsOfTftarget2REV1, and MsOffTarget3FWD1 and MsOffTarget3REV1, respectively (see Supplementary Table 1 for primer sequences). The resulting amplicons were then cloned as described above and clones from 8–12 colonies from each genotype and for each possible off-target site were sequenced to assess for mutations.

### Morphological Evaluations

For evaluation of plant height, branch number and internode length, two separate trials were carried out 30 days (pre-flowering) and 60 days (post-flowering) following the second cut. For the trial carried out 30 days following cutting, five biological replicates of well-watered SPL8-gRNA1-1 (∼75% GEF) and SPL8-gRNA1-2 (∼50% GEF), respectively, as well as five biological replicates for each of three independent EV control genotypes (15 biological replicates total), were analyzed in each case. For the trial carried out 60 days following cutting, ten biological replicates of well-watered SPL8-gRNA1-1 and -2 genotypes, respectively, as well as ten biological replicates for each of three independent EV control genotypes (30 biological replicates total), were assessed. Branch number was determined by counting the total number of branches per plant, while the longest shoot of each plant was utilized to measure plant height and the length of the longest internode.

The onset of flowering was recorded when petals first began to emerge from flower buds following the second cut. Subsequent to flowering (60 days following cutting), all aboveground biomass was removed and weighed to determine fresh weight (FW), and was then dried at 65°C for 1 week and weighed to determine dry weight (DW). Root length was evaluated by measuring the longest primary root, and root DW was resolved following drying at 65°C for 1 week.

Leaf length, width and area were measured using the middle leaflet of the third fully expanded trifoliate leaf from the shoot tip. Measurements were made 30 days following the second cut using five biological replicates (derived from vegetative stem cuttings) of each SPL8-gRNA1 genotype, and five biological replicates from each of three independent EV transformants, with three leaves assessed per plant. Leaf area was determined using the Petiole plant leaf area meter app (version 2.0.1^5^) and leaf dry weight was established after drying at 80°C for 24 h. Specific leaf area (SLA) was calculated by dividing the leaf area by dry weight in each case.

### Drought Treatment

Prior to the commencement of drought treatment, a volumetric soil moisture content of 50% was established in each pot using a ML3 ThetaKit soil moisture meter with 6 cm probes (Hoskin Scientific Ltd., Burnaby, BC, Canada), after which time water was withheld and volumetric soil water contents were measured daily until they reached approximately 1% (approximately 2–3 weeks). Assessments of water deficit symptoms were carried out on the basis of soil moisture contents rather than days following the withholding of water to minimize possible effects due to possible variation in plant water uptake. Subsequently, plants were re-watered normally for approximately 2 weeks and survival was assessed. Furthermore, volumetric soil moisture content was noted at the first instance of stress symptoms, which initially included the appearance of wilted or drooping leaves and shoots, in both edited and EV genotypes. Well-watered plants were utilized as controls. Six biological replicates of SPL8-gRNA1-1 (∼75% GEF) and SPL8-gRNA1-2 (∼50% GEF) genotypes, as well as three independent EV control genotypes (18 biological replicates total), were utilized in each experimental set. The withholding of water was initiated 14 days following the second cut.

### Measurement of Relative Water Content

Plant water status was estimated by measuring the relative water contents (RWC) of third fully expanded trifoliate leaves from six biological replicates, with two leaves assessed per replicate plant, of SPL8-gRNA1-1 and -2 genotypes, respectively, as well as 18 biological replicates of EV controls (six biological replicates from three independent transformants). Measurements were carried out as described previously (Barrs and Weatherley, 1962) when soil moisture contents reached approximately 50% (well-watered), 12–16% (moderate drought) and 7% (severe drought). In brief, the fresh weight (FW) of trifoliate leaves was documented immediately following detachment, turgid weights (TW) were determined after submersing petioles in water in an enclosed 1.5 mL microcentrifuge tube for 4 h, and dry weights (DW) were resolved by drying turgid leaves at 80°C overnight. Relative water content (%) was then calculated as [(FW-DW)/(TW-DW)] × 100.

### Detached Leaf Water Loss Assays

Detached leaf water loss assays were carried out essentially as described previously (Singer et al., 2021b) using fully expanded trifoliate leaves (third from the shoot tip) from eight biological replicate plants of each SPL8-gRNA1 genotype (two leaves per plant; sixteen replicates total for each) and seventeen biological replicate control plants (derived from three independent transformants with two leaves sampled per plant; thirty-four replicates total) under well-watered conditions. Assays were carried out by weighing leaves immediately upon harvest (W_initial_), placing them on a benchtop, and then weighing every 30 min for 240 min. The rate of water loss was calculated as (W_initial_ – W_atparticulartime_/W_initial_) × 100.

### Assessment of Photosynthetic Parameters

Light-saturated photosynthetic rate (Asat), stomatal conductance (*g*_s_), and transpiration rate (E) were evaluated using a LI-6800 (LI-COR Inc., Lincoln, NE, United States). The middle leaflet of a third fully expanded trifoliate leaf was used for measurements on four biological replicate plants of each SPL8-gRNA1 genotype, and four biological replicate plants of each of three EV control genotypes (twelve replicates total). All assessments were conducted between 10:30 am and 12:00 pm under greenhouse conditions. Measurements were made on one set of plants grown under well-watered conditions (soil moisture content of approximately 50%) and another subjected to drought treatment (average soil moisture content of approximately 11%). Within the chamber, light intensity was held at 1,500 μmol m^–2^ s^–1^, relative humidity at 50%, CO_2_ level at 410 μmol CO_2_/mol air, and heat exchanger temperature was set to 25°C. Leaflets were allowed to stabilize for 1.5 min within the chamber prior to measurement. All measurements were adjusted for leaf area, which was determined using the Petiole Pro plant leaf area meter app (version 1.4.15^6^).

### Crossing of Alfalfa Genotypes and Molecular Assessment of F_1_ Genotypes

Due to the outcrossing nature of alfalfa, we carried out manual reciprocal crosses between SPL8-gRNA1-1 (∼75% GEF) and the cultivar AC Blue J, as well as between SPL8-gRNA1-2 (∼50% GEF) and AC Blue J, using non-emasculated flowers. In each case, four distinct genotypes of AC Blue J were used along with eight biological replicates of each mutant genotype (derived from vegetative stem cuttings). A total of 32–64 seeds from each cross were sown and grown in the greenhouse as described in a previous section. Genomic DNA was harvested from a single leaf of each resulting F_1_ plant and PCR was conducted as described for T_0_ plants to screen for the presence of the transgene. T7E1 and ddPCR assays were carried out to assess editing frequencies at the gRNA1 target site using genomic DNA derived from F_1_ genotypes lacking a transgene as described previously for T_0_ plants.

### Statistical Analyses

Statistical differences between the means of SPL8-gRNA1 genotypes and EV controls were determined using student’s *t*-tests assuming unequal variance. Means were considered significantly different at *P* ≤ 0.05.

## Results

### Selection of *MsSPL8*-Specific gRNA Sequences

As a means of eliciting mutations in alfalfa *MsSPL8*, three gRNAs were designed to simultaneously target four *MsSPL8* alleles using CRISPR/Cas9 technology. These four alleles corresponded to MSAD_231217 and MSAD_231229 in the *M. sativa* genome sequence at the diploid level (CADL), or chr 8.2 83866861:83868757, chr 8.3 81917086:81918958; chr 8.4 80839466:80841286 and 80871132:80872948 from a tetraploid reference genome sequence (Chen et al., 2020). These four alleles exhibited 98.0% identity within their coding regions at the nucleotide level and 98.2% identity at the amino acid level. Using the freely available LegumeIP database and the *M. sativa* CADL genome (v1.0), the expression profiles of MSAD_231217 and MSAD_231229 in a selection of tissue types indicated that both transcripts were highly expressed in flowers, and to a lesser extent in elongated stems, while only low levels of expression were observed in roots, nodules and leaves (Supplementary Figure 2). The coding regions of the *MsSPL8* alleles possess three exons and two introns, and the predicted proteins incorporate a single SBP domain that is identical among *MsSPL8* alleles and also to that of *M. truncatula* Medtr8g005960. All three gRNAs were designed to target regions of the coding sequence within exon 1, and exhibited 100% complementarity to all four *MsSPL8* alleles. While gRNA1 and gRNA2 targeted regions upstream of the SBP domain, gRNA3 targeted a region within this domain (Figure 1A).

### Gene Editing Frequencies in SPL8-gRNA Alfalfa Genotypes at Population and Plant Levels

In order to target *MsSPL8* alleles using CRISPR/Cas9 in alfalfa, three pKSE401-based binary vectors bearing distinct gRNAs targeting all four *MsSPL8* alleles (SPL8-gRNA1, SPL8-gRNA2 and SPL8-gRNA3; Figure 1B) were generated. Each vector, along with an EV control consisting of pKSE401 without any gRNA sequence, was introduced into alfalfa, and PCR assessment of kanamycin-resistant genotypes provided confirmation for 26, 12, 12 and 17 independent SPL8-gRNA1, SPL8-gRNA2, SPL8-gRNA3 and EV transgenic genotypes, respectively.

**FIGURE 2:**
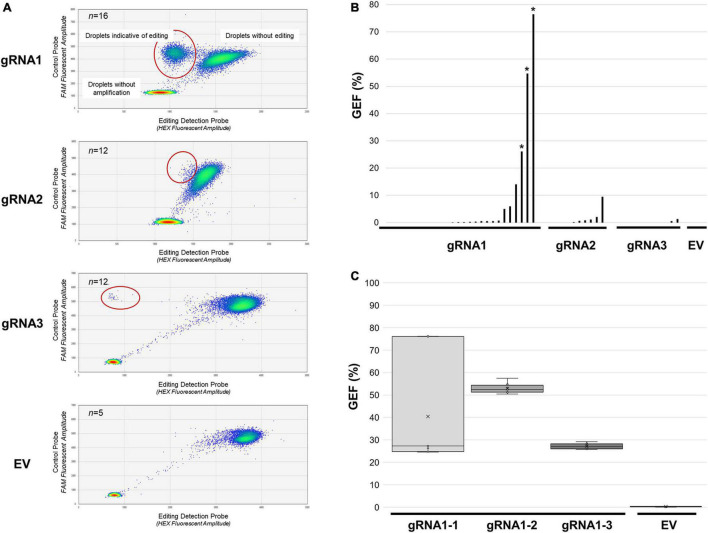
Assessment of *MsSPL8* editing frequencies in transgenic plants using GEF-ddPCR. **(A)** FAM (control probe) and HEX (editing probe) fluorescence following GEF-ddPCR of SPL8-gRNA transgenic genotypes, as well as a selection of EV controls. Heat maps indicate droplets that were not amplified, as well as those that bound both probes (not edited) and those that only bound the control probe (potentially edited, circled in red). **(B)** GEF of each transgenic genotype assessed using GEF-ddPCR. Asterisks denote genotypes that were selected for screening of separate branches [shown in panel **(C)**]. **(C)** Evaluation of GEF in eight distinct branches from three independent edited SPL8-gRNA1 genotypes, as well as EV controls. Blocks represent the upper and lower quartiles of the values obtained, x denotes median values, and bars indicate maximum and minimum values. EV, empty vector controls, gRNA1, SPL8-gRNA1 genotypes, gRNA2, SPL8-gRNA2 genotypes, gRNA3, SPL8-gRNA3 genotypes.

In order to quantitatively assess the frequency of editing in our transgenic genotypes, GEF-ddPCR was carried out using genomic DNA from each of the confirmed independent transgenic SPL8-gRNA genotypes, as well as five independent EV negative control genotypes (Figures 2A,B). Of the transgenic genotypes assessed, 10 of 26 (38.5%) SPL8-gRNA1 genotypes, 5 of 12 (41.7%) SPL8-gRNA2 genotypes, and 2 of 12 (16.7%) SPL8-gRNA3 genotypes exhibited GEF ≥ 0.5%, which was chosen as the threshold indicating the presence of at least a small number of edits in a sample. None of the five EV controls exhibited a GEF above 0.2%. In the case of SPL8-gRNA1, SPL8-gRNA2 and SPL8-gRNA3 genotypes, GEF of up to 76.5, 9.5, and 1.3%, respectively, were observed (Figure 2B). Since gRNA1 appeared to exhibit superior editing efficiency compared to gRNA2 and gRNA3, we selected three independent T_0_ transgenic SPL8-gRNA1 genotypes with relatively high GEF [labeled SPL8-gRNA1-1 (76.5% GEF), SPL8-gRNA1-2 (54.7% GEF), and SPL8-gRNA1-3 (26.1% GEF)] for subsequent experiments.

Since there are four *MsSPL8* alleles in alfalfa, a GEF of less than approximately 25% indicates probable mosaicism for editing events within a single plant. To evaluate whether edited genotypes exhibiting a GEF above 25% were also mosaics, genomic DNA was isolated from leaves derived from eight distinct branches of SPL8-gRNA1-1, SPL8-gRNA1-2 and SPL8-gRNA1-3 genotypes, respectively, and subjected to GEF-ddPCR. While SPL8-gRNA1-1 exhibited a rather wide range of GEF among branches (between 24.6% and 76.2%), SPL8-gRNA1-2 and -3 displayed much narrower ranges (between 50.4–57.5% and 25.7–29.2%, respectively) (Figure 2C). In an attempt to minimize variation in GEF in the SPL8-gRNA1-1 genotype, all subsequent experiments were carried out using plants derived from vegetative stem cuttings of the branch exhibiting a 76.2% GEF in the original T_0_ transformant.

### Validation of NHEJ-Derived Mutations and Specificity in SPL8-gRNA1 Alfalfa Genotypes

**FIGURE 3:**
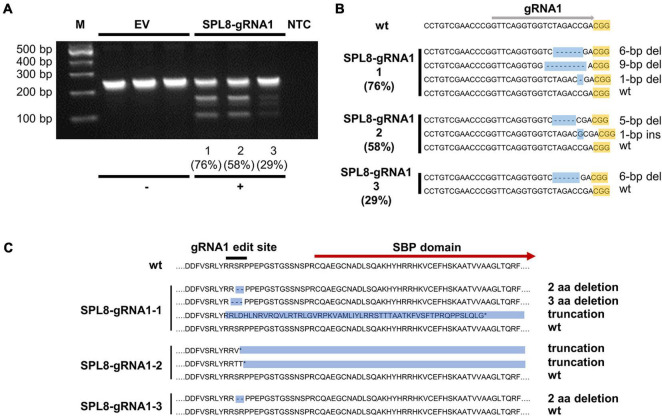
Confirmation of *MsSPL8* editing in SPL8-gRNA1 genotypes. **(A)** T7E1 assay results from three empty vector (EV) control genotypes and SPL8-gRNA1-1, SPL8-gRNA1-2 and SPL8-gRNA1-3 genotypes. While genotypes with no alterations within the *MsSPL8* gRNA1 target region show a single band, those with genetic modifications within the gRNA1 target region exhibit multiple bands. Percentages indicate gene editing frequencies determined *via* GEF-ddPCR. **(B)** Nucleotide sequences surrounding the *MsSPL8* gRNA1 target region from untransformed (wt), SPL8-gRNA1-1, -2 and -3 genotypes as determined by Sanger sequencing. All results are representative of all genetic variants observed within each genotype following the sequencing of 21–33 clones. Percentages indicate gene editing frequencies determined *via* GEF-ddPCR. **(C)** Amino acid sequences of *MsSPL8* gRNA1 target region from untransformed (wt), SPL8-gRNA1-1, -2 and -3 genotypes.

To provide further confirmation of NHEJ-mediated gene editing in *MsSPL8* alleles within SPL8-gRNA1 genotypes, we carried out T7E1 mismatch cleavage assays on genomic DNA isolated from SPL8-gRNA1-1, -2 and -3 plants, as well as three independent EV controls. Single bands of approximately 250-bp were observed in all EV controls, which indicates that no indels were present in any *MsSPL8* allele within the amplified region. Conversely, all three SPL8-gRNA1 genotypes assessed exhibited multiple bands, including uncut amplicon of approximately 250-bp, as well as fragments consistent with the presence of an indel at the gRNA1 target site (approximately 150-bp and 100-bp; Figure 3A), which confirms the presence of edits at the target site in these genotypes.

To identify the precise genetic changes that occurred at the gRNA1 target site in edited alfalfa genotypes, we subsequently cloned and sequenced a region encompassing the gRNA1 target site from SPL8-gRNA1-1 (76.4% GEF), SPL8-gRNA1-2 (57.5% GEF) and SPL8-gRNA1-3 (29.2% GEF) genotypes, as well as untransformed N4.4.2. Clones from between 21 and 33 colonies were sequenced in every case. No clone derived from N4.4.2 exhibited any genetic variation within the *MsSPL8* gRNA1 target region. In contrast, in the case of SPL8-gRNA1-1, we detected three distinct mutations 2- to 4-bp upstream of the gRNA1-associated PAM (a 6-bp deletion, a 9-bp deletion and a 1-bp deletion) along with wild-type sequence. In SPL8-gRNA1-2, we observed two distinct mutations at this same site (a 1-bp insertion and a 5-bp deletion) along with wild-type sequence. In SPL8-gRNA1-3, a single mutation at the predicted gRNA1 cut site (6-bp deletion) and wild-type sequence were observed (Figure 3B). While the 6-bp and 9-bp deletions would result in missing SR or RSP amino acid residues just upstream of the SBP domain, the 1-bp deletion, 1-bp insertion and 5-bp deletion would lead to a frameshift and the presence of premature stop codons. All of these stop codons are located either upstream of, or within, the SBP domain (Figure 3C).

To assess for possible off-target effects in the SPL8-gRNA1 alfalfa genotypes, we initially screened the *M. sativa* CADL v0.95 genome sequence with that of gRNA1 using the Cas-Offinder program. No matches were identified with a 1-nt mismatch and a 0-, 1- or 2-nt RNA bulge, or with two mismatches and 0- or 1-nt RNA bulge. However, possible off-target sites were identified with two single nucleotide mismatches and a 2-nt RNA bulge, and three single nucleotide mismatches and a 1-nt RNA bulge. To confirm that off-target mutations had not occurred in our three selected SPL8-gRNA1 genotypes, we cloned and sequenced three regions that spanned possible off-target sites for this gRNA (Supplementary Figure 1) from SPL8-gRNA1-1, -2 and -3, as well as untransformed N4.4.2 controls. No genetic variation was noted within the three possible off-target regions in any of the 8–12 clones sequenced for each genotype.

Due to a lack of a frameshift mutation in the SPL8-gRNA1–3 genotype, all further experiments focused on SPL8-gRNA1-1 and SPL8-gRNA1-2 genotypes.

### SPL8-gRNA1 Genotypes Exhibit Morphological Phenotypes That Are Distinct From EV Genotypes

**FIGURE 4:**
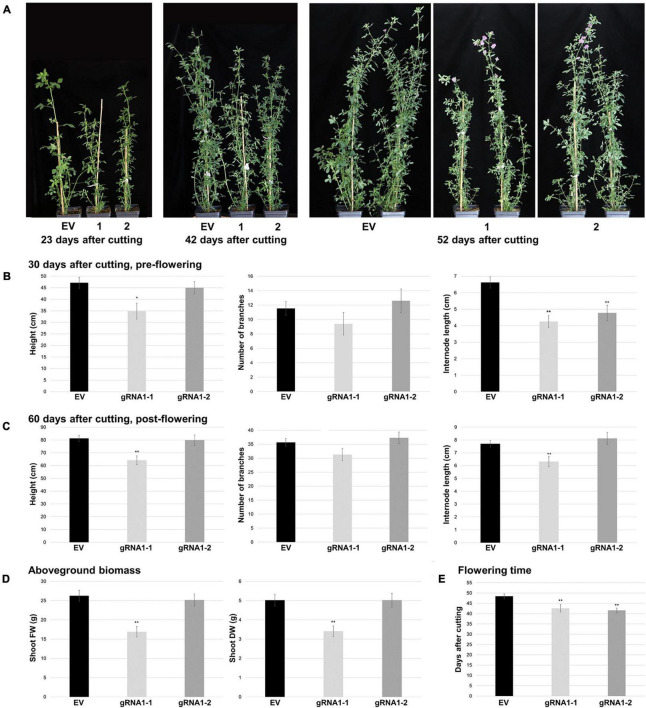
Aboveground growth characteristics of SPL8-gRNA1 and EV control genotypes. **(A)** Representative images are of edited SPL8-gRNA1-1 (1) and SPL8-gRNA1-2 (2) genotypes, as well as EV controls, 23, 42 and 52 days following cutting. **(B,C)** Graphs depict plant height, total number of branches and internode length 30 **(B)** and 60 **(C)** days after cutting. **(D)** Shoot fresh weight (FW) and dry weight (DW) 60 days after cutting when all plants were flowering. **(E)** Number of days following cutting at which plants began to flower. For all graphs, each block represents the mean of five SPL8-gRNA1-1 (gRNA1-1), five SPL8-gRNA1-2 (gRNA1-2) and fifteen EV (five each of three independent transformants) biological replicates **(B)** or ten SPL8-gRNA1-1 (gRNA1-1), ten SPL8-gRNA1-2 (gRNA1-2) and thirty EV (ten each of three independent transformants) **(C–E)** biological replicates derived from stem cuttings, while bars denote standard errors. Asterisks denote means that are significantly different from EV controls as determined by 2-tailed student’s *t*-tests assuming unequal variance (*P ≤ 0.05; **P ≤ 0.01).

To determine whether SPL8-gRNA1-1 and SPL8-gRNA1-2 genotypes differed from EV plants in terms of their morphology, we assessed various aboveground and belowground characteristics under well-watered conditions. Although no changes in the number of branches were observed prior to flowering, both SPL8-gRNA1-1 and SPL8-gRNA1-2 genotypes displayed decreases in internode length (35.7% and 27.9% relative decreases, respectively), and SPL8-gRNA1-1 exhibited a significant reduction in plant height (26.1% relative decrease), compared to EV controls (Figures 4A,B). Similarly, post-flowering, significant reductions in plant height and internode length (Figures 4A,C), shoot fresh weight (FW) and dry weight (DW) (Figure 4D), and root length and DW (Supplementary Figure 3) were noted in SPL8-gRNA1-1, but not SPL8-gRNA1-2, compared to EV controls. As was the case pre-flowering, no significant differences were observed between either SPL8-gRNA1 genotype and EV controls with respect to branch number post-flowering (Figure 4C). Taken together, these results suggest that shoot and root growth phenotypes in SPL8-gRNA1 genotypes may be gene dosage-dependent, with the genotype exhibiting an approximately 75% GEF in *MsSPL8* alleles exhibiting more severe alterations than that with an approximately 50% GEF.

**FIGURE 5:**
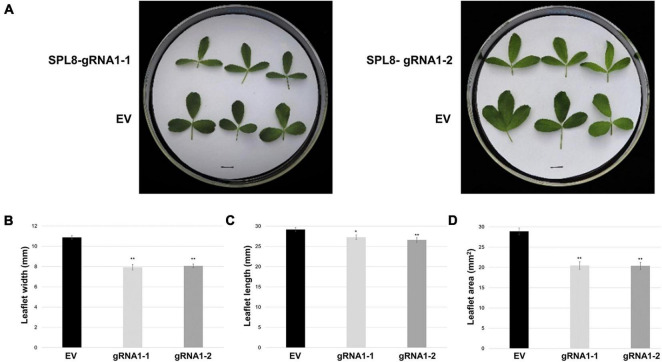
Leaf characteristics of SPL8-gRNA1 and EV control genotypes. **(A)** Representative images of trifoliate leaves (third from the shoot tip) from edited SPL8-gRNA1-1 (mutations in three *MsSPL8* alleles) and SPL8-gRNA1-2 (mutations in two *MsSPL8* alleles) genotypes, as well as empty vector (EV) controls. Scale bars = 1 cm. **(B–D)** Measurements of the width, length and area of the middle leaflet of trifoliate leaves (third from the shoot tip) at 30 days after cutting. Each block represents the mean of three leaflets from five biological replicates of each SPL8-gRNA1 genotype, and five biological replicates from each of three independent EV transformants (fifteen replicates total), while bars denote standard errors. Asterisks denote means that are significantly different from EV controls as determined by 2-tailed student’s *t*-tests assuming unequal variance (*P ≤ 0.05; **P ≤ 0.01).

In addition to growth phenotypes, both edited SPL8-gRNA1 genotypes exhibited significant reductions in the time required for flowering onset compared to EV controls, with SPL8-gRNA1-1 and -2 flowering on average 42.6 and 41.6 days after cutting and EV genotypes flowering on average 48.5 days after cutting (Figures 4A,E). Furthermore, leaf characteristics were consistently altered in both SPL8-gRNA1-1 and SPL8-gRNA1-2 genotypes compared to EV controls (Figure 5A). Specifically, SPL8-gRNA1-1 and SPL8-gRNA1-2 exhibited significant reductions in leaf width (27.1% and 25.9% relative decreases, respectively; Figure 5B), leaf length (6.5% and 8.8% relative decreases, respectively; Figure 5C) and leaf area (29.2% and 29.4% relative decreases, respectively; Figure 5D) compared to EV genotypes.

### SPL8-gRNA1 Genotypes Exhibit Enhanced Drought Resilience Compared to EV Controls

**FIGURE 6:**
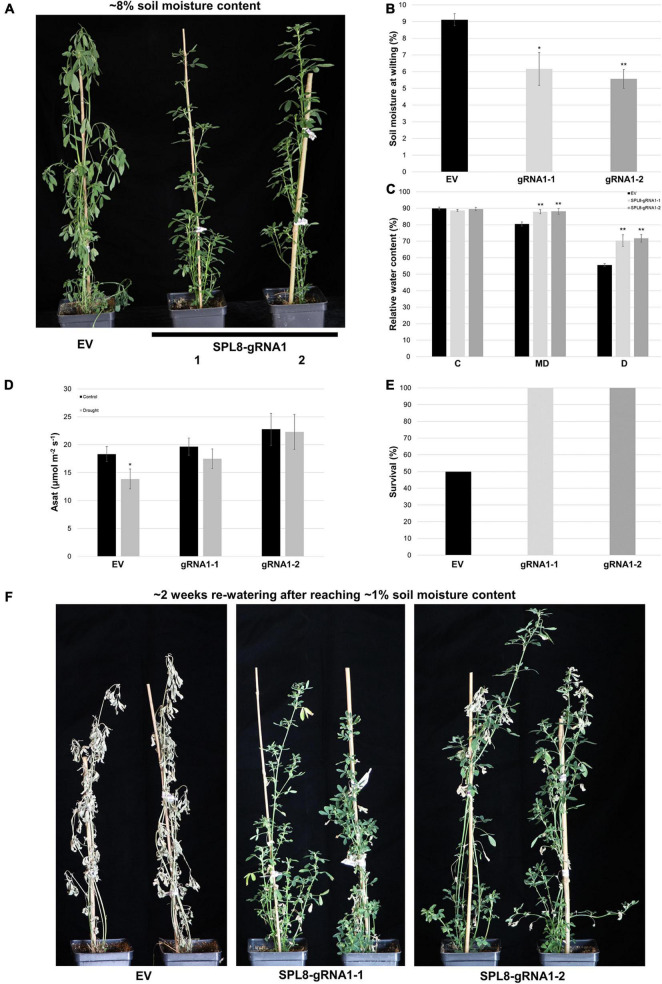
Response of SPL8-gRNA1 and EV genotypes to drought stress. **(A)** Representative images of SPL8-gRNA1-1, SPL8-gRNA1-2 and EV genotypes after withholding water until volumetric soil moisture levels reached approximately 8%, 11 days after initiating the withholding of water. **(B)** Soil moisture level at which SPL8-gRNA1 and EV genotypes began to wilt. **(C)** Relative water content (RWC) of third fully expanded trifoliate leaves assessed when soil moisture contents were at approximately 50% [control (C)], 14% [moderate drought (MD)] and 7% [drought (D)]. Blocks in each graph consist of the mean value of six SPL8-gRNA1-1 (gRNA1-1), six SPL8-gRNA1-2 (gRNA1-2) and eighteen EV (six each of three independent transformants) biological replicates derived from stem cuttings. **(D)** Light-saturated photosynthetic rate (Asat) of middle leaflets from third fully expanded trifoliate leaves under well-watered (soil moisture content of approximately 50%) and drought (average soil moisture of approximately 11%) conditions. Measurements were adjusted for leaf area. Blocks represent the mean value of leaflets from four SPL8-gRNA1-1, four SPL8-gRNA1-2, and twelve EV (four each of three independent transformants) biological replicate plants derived from stem cuttings. **(E)** Proportion of plants that survived following 2 weeks of re-watering after allowing soil moisture contents to reach 1%. Blocks consist of the mean value of six SPL8-gRNA1-1 (gRNA1-1), six SPL8-gRNA1-2 (gRNA1-2) and eighteen EV (six each of three independent transformants) biological replicates derived from stem cuttings. **(F)** Representative images of SPL8-gRNA1-1, SPL8-gRNA1-2 and EV genotypes following approximately 2 weeks of re-watering once plants reached 1% soil moisture contents. For all graphs, bars denote standard errors and asterisks denote means that are statistically different in SPL8-gRNA1 genotypes compared to EV genotypes **(B,C)** or under drought compared to well-watered conditions **(D)** as determined by 1-tailed **(D)** or 2-tailed **(B,C)** *t*-tests assuming unequal variance (*P ≤ 0.05; **P ≤ 0.01).

In order to evaluate whether genotypes with mutations in *MsSPL8* alleles were able to better withstand drought stress than EV genotypes, we withheld water from plants with an initial soil moisture content of approximately 50% and assessed the soil moisture content at which they began to exhibit signs of stress, including wilted or drooping leaves and shoots (Figure 6A). Both SPL8-gRNA1-1 and SPL8-gRNA1-2 genotypes wilted at a significantly lower soil moisture content (an average of 6.2% and 5.6% volumetric soil moisture content, respectively) than EV controls (an average of 9.1% volumetric soil moisture) (Figures 6A,B). In line with this, while leaf RWC did not differ between genotypes under well-watered conditions, both SPL8-gRNA1 genotypes exhibited significant increases in leaf RWC under moderate (approximately 14% volumetric soil moisture content) and severe (approximately 7% volumetric soil moisture content) drought conditions (Figure 6C), which indicates that the edited genotypes were better able to retain water in their leaves in the face of water deficit. This was also apparent in the physical appearance of plants at soil moisture levels of approximately 8%, at which point EV control plants consistently displayed obvious water-deficit symptoms, while SPL8-gRNA1 plants remained visibly unaffected (Figure 6A). Furthermore, while no significant difference was noted in light-saturated photosynthetic rate between EV and SPL8-gRNA1 genotypes under well-watered conditions, only EV genotypes displayed a significant reduction in light-saturated photosynthetic rate under drought compared to control conditions (Figure 6D). This suggests that drought treatment negatively impacted photosynthesis in EV genotypes, but not SPL8-gRNA1 genotypes. Finally, when plants were allowed to reach a soil moisture content of 1% and were then re-watered for approximately 2 weeks, only 50% of EV genotypes survived, while all SPL8-gRNA1 genotypes flourished, exhibiting necrosis in only a small number of leaves (Figures 6E,F), which further illustrates the enhanced drought tolerance of the edited genotypes in this study.

**FIGURE 7:**
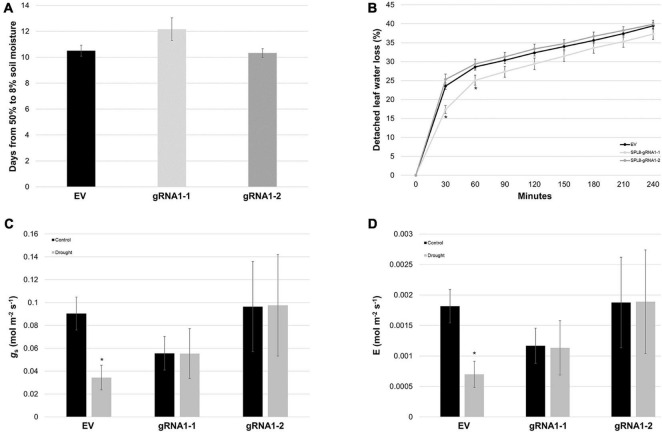
Water loss-related traits in SPL8-gRNA1 and EV genotypes. **(A)** Number of days required for volumetric soil moisture contents to decrease from 50 to 8% following the withholding of water. Blocks denote the mean value of six SPL8-gRNA1-1, six SPL8-gRNA1-2 and eighteen EV (six each of three independent transformants) biological replicates derived from stem cuttings. **(B)** Rates of water loss from detached leaves derived from well-watered plants. Blocks denote the mean value of twelve SPL8-gRNA1-1 (two leaves from six plants), twelve SPL8-gRNA1-2 (two leaves from six plants) and thirty-six EV (twelve leaves from three independent transformants) biological replicates. **(C,D)** Stomatal conductance (*g*_s_) and transpiration rate (E) of middle leaflets from third fully expanded trifoliate leaves under well-watered (soil moisture content of approximately 50%) and drought (average soil moisture content of approximately 11%) conditions. Measurements were adjusted for leaf area and blocks represent the mean value of leaflets from four SPL8-gRNA1-1, four SPL8-gRNA1-2, and twelve EV (four each of three independent transformants) biological replicate plants derived from stem cuttings. For all graphs, bars denote standard errors and asterisks denote means that are statistically different in SPL8-gRNA1 genotypes compared to EV genotypes **(B)** or under drought compared to well-watered conditions **(C,D)** as determined by 1-tailed **(C,D)** or 2-tailed **(B)** *t*-tests assuming unequal variance (*P ≤ 0.05). gRNA1-1, SPL8-gRNA1-1; gRNA1-2, SPL8-gRNA1-2.

To determine whether differences in water use/loss were involved in the superior drought tolerance of SPL8-gRNA1 genotypes, we first assessed the rate at which soil moisture was lost from pots bearing different genotypes, and found no significant differences among genotypes (Figure 7A). Similarly, while SPL8-gRNA1-1 plants exhibited a significant decrease in detached leaf water loss rates compared to EV plants at early time points, no significant differences were observed at subsequent time points, or between SPL8-gRNA1-2 and EV plants (Figure 7B). Furthermore, while no significant differences were apparent in stomatal conductance or transpiration rate between SPL8-gRNA1 and EV genotypes either under well-watered or drought conditions, EV genotypes, but not SPL8-gRNA1 genotypes, exhibited significant reductions in both stomatal conductance and transpiration rate under drought compared to control conditions (Figures 7C,D). Taken together, these results suggest that unlike EV genotypes, SPL8-gRNA1 genotypes did not respond to the same level of drought stress by reducing stomatal conductance and transpiration rate, and that traits related to water use and/or water loss were not solely responsible for the improvement of SPL8-gRNA1 genotypes in terms of their ability to withstand water deficit.

### Generation of Non-transgenic Edited F_1_ Genotypes

Heritability of the *MsSPL8* edits was evaluated by crossing SPL8-gRNA1-1 and SPL8-gRNA1-2 genotypes, respectively, with several genotypes of the cultivar AC Blue J. A total of 202 seedlings were established from the crosses, and PCR analysis demonstrated that 23.8% of them lacked a transgene. Furthermore, T7E1 and ddPCR assays indicated that of the transgene-negative plants, 77.1% bore an edit in at least one *MsSPL8* allele (Table 1). None of the AC Blue J plants assessed exhibited a transgene or editing of this gene in any case, and GEF of 74.5% and 55.7% were confirmed for SPL8-gRNA1-1 and SPL8-gRNA1-2 genotypes, respectively (Table 1 and Supplementary Figure 4).

**TABLE 1:** Molecular evaluation of F_1_ genotypes derived from crosses between SPL8-gRNA1 and AC Blue J genotypes.

Cross	Total plants	Transgene-negative plants	GEF				
			∼0%	∼25%	∼50%	∼75%	∼100%
SPL8-gRNA1-1 (F) × AC Blue J (M)	60	10	0	4	5	1	0
SPL8-gRNA1-2 (F) × AC Blue J (M)	56	8	0	6	1	0	1
AC Blue J (F) × SPL8-gRNA1-1 (M)	55	20	8	6	6	0	0
AC Blue J (F) × SPL8-gRNA1-2 (M)	31	10	3	6	1	0	0
Total	202	48	11	22	13	1	1

*F, female parent; M, male parent.*

Intriguingly, with the exception of one genotype that exhibited a GEF of 34%, all transgene-negative seedlings fell into one of five GEF classes: 0% (0–0.6% GEF), 25% (24.0–28.7% GEF), 50% (48.7–53.2% GEF), 75% (74.5%) and 100% (99.9%). In both instances where SPL8-gRNA1 genotypes acted as the recipient for AC Blue J pollen, all transgene-negative plants possessed an edit in *MsSPL8*. Conversely, where AC Blue J was utilized as the recipient for SPL8-gRNA1-1 and SPL8-gRNA1-2 pollen, respectively, 40.0% and 30.0% of transgene-negative plants bore no edits (Table 1 and Supplementary Figure 4). Of the transgene-negative edited genotypes overall, 29.7% bore no edit, 59.5% possessed a GEF of approximately 25%, 35.1% possessed a GEF of approximately 50%, 2.7% possessed a GEF of approximately 75% and 2.7% possessed a GEF of 100% (Table 1 and Supplementary Figure 4). The genotypes with GEF of approximately 75% and 100% were derived from crosses where AC Blue J was utilized to pollinate SPL8-gRNA1-1 and SPL8-gRNA1-2, respectively, which suggests that a low level of selfing likely took place in the SPL8-gRNA1 genotypes. Taken together, these results confirm that edited genotypes lacking a transgene can be achieved in the next generation, and that editing was present in a range of allelic combinations.

## Discussion

As a means of further understanding the role of *MsSPL8* in plant development and abiotic stress response, and concomitantly generating transgene-free germplasm with reduced MsSPL8 activity, we utilized CRISPR/Cas9 technology to simultaneously edit multiple *MsSPL8* alleles in alfalfa. Although the CRISPR/Cas9 platform has proven extremely effective in many plant species to date, including those with polyploid genomes (Morineau et al., 2017; Liu et al., 2018; Huang et al., 2020), it has only very recently begun to gain traction in alfalfa (Gao et al., 2018b; Chen et al., 2020; Wolabu et al., 2020; Bottero et al., 2021; Curtin et al., 2021). The first attempt at utilizing this technique in this species involved the targeting of another *SPL* gene (*MsSPL9*), and while successful edits at the target site were detected, they occurred with extremely low frequencies of up to approximately 2.2% of alleles within the tissue tested (Gao et al., 2018b). As a result of the high level of mosaicism in these cases, phenotypic alterations were not observed. However, subsequent studies have led to substantially higher editing frequencies and consequent phenotypic alterations in alfalfa, with up to 100% allelic mutation frequency observed in the first generation when targeting the *M. sativa* stay-green (*MsSGR*), phytoene desaturase (*MsPDS*) and *PALM1* genes (Chen et al., 2020). While the use of a multiplex gRNA CRISPR/Cas9 system was required to achieve high editing frequencies in alfalfa in certain instances (Wolabu et al., 2020; Bottero et al., 2021), this has not always been the case (Chen et al., 2020). In the current study, we were able to achieve high frequencies of indels in *MsSPL8* alleles in the first generation (with up to three of four alleles mutated) with a single gRNA (Figures 2B, 3B,C), and in a manner that was homogeneous in at least a proportion of genotypes (Figure 2C). In addition, this was achieved in a manner that was highly specific with no off-target mutations observed in the selection of sites analyzed (Supplementary Figure 1).

As has been reported elsewhere in the literature for both alfalfa and other plants species (Bruegmann et al., 2019; Ren et al., 2019; Wolabu et al., 2020; Zhang et al., 2020), we noted a considerable difference in editing efficiencies among gRNAs used in this study (Figure 1A), with gRNA1 exhibiting much higher editing frequencies than gRNA2 and gRNA3 (Figures 2, 3). While some progress has been made in terms of identifying gRNA characteristics that are important for cleavage efficiency in animal systems (Doench et al., 2014), a sound understanding of this issue specifically in plants remains largely elusive. Currently existing gRNA ranking prediction algorithms for plants are based on experiments in animals, and it has been shown that there is little correlation among rankings achieved using these prediction tools and effectiveness *in vivo* (Naim et al., 2020). It has been suggested that GC content and the proportion of purines in the last four positions of the gRNA may be important for gRNA efficiency in plants (Bruegmann et al., 2019; Ren et al., 2019); however, these parameters were not responsible for the higher effectiveness of gRNA1 in the current study since all three gRNAs bore a GC content of 55% and gRNA2 possessed the highest number of purines near the 3′ terminus (three of four nucleotides) (Figure 1A). Furthermore, although the particular DNA strand targeted by a gRNA has also been proposed to play a role in its activity in certain studies (Bruegmann et al., 2019), this has not always been a factor (Doench et al., 2014) and did not appear to be the case in the current study since both gRNA1 and gRNA2 targeted the same strand (Figure 1A).

Highly similar phenotypes were apparent in both of the edited genotypes analyzed (derived from independent transformation events), including reductions in internode length and leaf size, as well as early flowering (Figures 4, 5). Plant height, shoot and root biomass, and root length were also significantly reduced in SPL8-gRNA1-1, but not SPL8-gRNA1-2, compared to EV controls (Figure 4 and Supplementary Figure 3), which reflects the mutational dosage in the two genotypes. SPL8-gRNA1-1 possessed a single *MsSPL8* allele bearing a frameshift mutation, two alleles bearing mutations that would result in two and three amino acid deletions, respectively, and a wild-type allele. Conversely, SPL8-gRNA1-2 bore frameshift mutations in two *MsSPL8* alleles along with two wild-type *MsSPL8* alleles (Figure 3C). While it is currently unclear whether the deletions present in the two *MsSPL8* alleles in SPL8-gRNA1-1 would lead to complete inactivation of the SPL8 protein, given the proximity of the cleavage site to the SBP box it is certainly feasible that they could negatively impact activity. As such, it is not entirely surprising that the edited genotypes displayed a gene dosage effect in line with the number of alleles that bore mutations in each case, with the genotype bearing mutations in three *MsSPL8* alleles (SPL8-gRNA1-1) exhibiting phenotypic alterations that were more profound than the genotype bearing mutations in only two *MsSPL8* alleles (SPL8-gRNA1-2). While this type of additive effect has been noted previously following editing in other polyploid plant species such as *Camelina sativa* (Morineau et al., 2017), *Brassica napus* (Huang et al., 2020) and *Triticum aestivum* (Li et al., 2021), this is the first instance in which it has been demonstrated in alfalfa. However, this phenomenon may not occur in every instance, and a previous study where CRISPR/Cas9 was used to successfully target *MsPDS* and *MsPALM1* in alfalfa found that an altered phenotype was only observed when all four alleles had been mutated simultaneously (Chen et al., 2020). This discrepancy may be attributable to the particular gene target and/or the resulting phenotypic change that occurs following editing, although further research will be required to unravel such distinctions in plants fully.

Interestingly, the pleiotropic phenotypes observed in MsSPL8-gRNA1 genotypes did not parallel the expression patterns noted previously for this gene, whereby expression was primarily detected in flowers and elongated stems, with little expression in leaves or roots (Supplementary Figure 2). This phenomenon is not specific to alfalfa, and a similar discrepancy has been observed previously in Arabidopsis, whereby root phenotypes were observed in *spl8* mutants despite an apparent lack of expression in this tissue (Zhang et al., 2007). Although similar *SPL8* expression patterns have been observed in Arabidopsis and *Medicago* spp. (Xing et al., 2013; Gou et al., 2018; Supplementary Figure 2), there appears to be considerable distinctions between the functions of *SPL8* homologs across species (Unte et al., 2003; Zhang et al., 2007; Xing et al., 2013; Gou et al., 2018). Unlike *MsSPL8* in alfalfa, Arabidopsis *SPL8* appears to be mainly involved in the regulation of male and female fertility, as well as root growth, with very few apparent roles in aboveground vegetative growth (Unte et al., 2003; Zhang et al., 2007; Xing et al., 2013). In addition, while *SPL8* does not appear to be regulated by miR156 in Arabidopsis (Xing et al., 2010), it is a target of this miRNA in alfalfa (Feyissa et al., 2021), which suggests that some level of evolutionary divergence has occurred in this gene among plant species. However, despite these possible functional and regulatory disparities among species, both Arabidopsis and alfalfa *SPL8* have been suggested to function, at least in part, through the transcriptional regulation of genes involved in phytohormone biosynthesis or signaling (Zhang et al., 2007; Xing et al., 2013; Gou et al., 2018), although their precise functions remain to be unraveled in full. In line with this, *M. truncatula spl8* mutant plants were found to accumulate higher GA_4_ levels in mature leaves and the shoot apical meristem than the wild-type control (Gou et al., 2018). In the current study, the phenotypes noted in the vegetative tissues of independent SPL8-gRNA1 genotypes were reminiscent of GA-deficient mutants, which tend to be dwarfed in stature with reductions in leaf expansion and stem elongation (Thomas and Sun, 2004; Figures 4, 5). Conversely, we also observed early flowering in our edited genotypes (Figure 4E), which is instead typical of mutants with an over-accumulation of GA (Huang et al., 1998). Although GA levels were not evaluated in the present study, Arabidopsis *SPL8* provides opposing roles in inflorescences and seedlings in the context of GA-dependent developmental processes (Zhang et al., 2007), which hints at the possibility that a similar phenomenon may be occurring in alfalfa. However, other mechanisms could also be involved in the observed phenotypes in SPL8-gRNA1 genotypes, and in depth transcriptomic assessments, as well as evaluations of GA levels, GA signaling and GA-related pathways, will be necessary to unravel these differences between species and studies in full.

While the reduced stature and internode length observed in SPL8-gRNA1 genotypes in the current study resembled RNAi genotypes in which other miR156-regulated *SPL* genes, including *SPL9* and *SPL13*, were down-regulated in alfalfa (Gao et al., 2018a; Hanly et al., 2020), our edited genotypes did not exhibit the increased branching, enhanced biomass or delayed flowering that are often seen in such genotypes (Figure 4). Similarly, the SPL8-gRNA1 genotypes in the current study did not closely resemble *MsSPL8* RNAi genotypes or the *M. truncatula spl8* mutant, which displayed increased branching and forage biomass, accelerated regrowth and no apparent change in flowering time or leaf size (Gou et al., 2018). It is not clear why this was the case; however, it is possible that differences in growth conditions (including potting mix, pot size, light intensity or temperature, for example), the background genotype utilized for the study, or the specific alleles targeted in RNAi and CRISPR/Cas9 studies may have played a role in these discrepancies.

A subset of miR156-targeted *SPL* genes in alfalfa have been found previously to lead to an increased ability to withstand abiotic stresses such as drought (*SPL13*, *SPL9*; *SPL8;* Arshad et al., 2017; Gou et al., 2018; Feyissa et al., 2019; Hanly et al., 2020), heat (*SPL13*; Matthews et al., 2019), salinity (*SPL8*; Gou et al., 2018) and/or flooding (*SPL13*; Feyissa et al., 2021) when down-regulated. Correspondingly, both SPL8-gRNA1 genotypes assessed in the present study exhibited improvements in their resilience to water deficit, with lower volumetric soil contents at the first sign of wilting, an improved ability to maintain RWC and photosynthetic rate under drought treatment, and higher survival following extreme drought stress (Figure 6). This improvement in drought tolerance across SPL8-gRNA1 genotypes does not appear to be a direct result of decreased water loss from their leaves (Figure 7). However, it is possible that reduced leaf size, which is known to be associated with superior drought tolerance in plants (Quan et al., 2016; Singer et al., 2021b), could be contributing to an overall reduction in water loss at the plant level. Intriguingly, enhancements in drought tolerance in RNAi alfalfa genotypes in which other *SPL* genes were down-regulated have been suggested to occur, at least in part, through the up-regulation of anthocyanin biosynthetic genes, thus resulting in an increased accumulation of this secondary metabolite and a consequent enhancement in ROS scavenging capacity (Gou et al., 2018; Feyissa et al., 2019; Hanly et al., 2020). While this may also be the case in our SPL8-gRNA1 genotypes, the precise mechanisms driving enhanced drought resilience remain to be elucidated.

Due to inbreeding depression, there is a need to outcross alfalfa, and as such, we carried out reciprocal crosses between our edited genotypes and a distinct cultivar bearing wild-type *MsSPL8* alleles (AC Blue J) in order to achieve progeny in which the transgenic cassette had been segregated out, but that possessed edits within *MsSPL8* alleles. Approximately 25% of the resulting seedlings assessed were found to lack the transgene, and of the transgene-free F_1_ genotypes, upward of 75% possessed an edit in at least one *MsSPL8* allele (Table 1 and Supplementary Figure 4), which confirms the previous finding that mutated alleles are stably inherited in this species (Chen et al., 2020). While the vast majority of genotypes fell into GEF classes of 25% and 50% (one of four and two of four *MsSPL8* alleles edited, respectively; Table 1 and Supplementary Figure 4), two genotypes exhibited GEF of 75% and 100% (three of four and four of four *MsSPL8* alleles edited, respectively). Since SPL8-gRNA1 genotypes were utilized as the female parent in both instances where unexpectedly high GEF were observed in offspring, it is highly likely that some level of selfing occurred in these genotypes. While self-pollination is not the predominant route for seed production in alfalfa typically, they are not self-incompatible, and selfing rates up to approximately 52% have been noted in this species (Riday et al., 2015).

In conclusion, we have successfully utilized CRISPR/Cas9 technology to edit up to three of four *MsSPL8* alleles in alfalfa in the first generation with a single gRNA, and up to 100% of alleles in the second generation. Despite the fact that at least one wild-type copy of *MsSPL8* remained in our first generation plants, we observed distinct morphological alterations in SPL8-gRNA1 genotypes, including small leaf size, reduced internode length and early flowering. These phenotypes differed from those observed previously in alfalfa RNAi genotypes targeting *MsSPL8*, which may be attributable to distinctions in growth conditions, the background genotype, or the specific alleles targeted between studies. The severity of phenotypic changes tended to be dependent on allelic mutation dose, and only genotypes with mutations in three of four *MsSPL8* alleles, but not those with mutations in two of four *MsSPL8* alleles, demonstrated significant reductions in plant height, shoot and root biomass, and root length. This is the first instance of such a phenomenon having been demonstrated in alfalfa. Finally, both SPL8-gRNA1 genotypes evaluated in the current study exhibited a superior ability to survive under water-deficit conditions compared to EV controls, which is an invaluable trait for a future of climate change, and is the first case in which an improvement in abiotic stress tolerance has been elicited in alfalfa using gene editing. Further in-depth examination of these edited genotypes will shed light on functional discrepancies among *SPL8* homologs from different species and/or genotypes, precise mechanisms driving improvements in drought resilience, and could potentially provide the capacity to target genes that function downstream of *MsSPL8* as a means of achieving abiotic stress tolerance without the reductions in biomass that are observed with high allelic mutation rates.

## Data Availability Statement

The original contributions presented in the study are included in the article/Supplementary Material, further inquiries can be directed to the corresponding author/s.

## Author Contributions

SS, AH, SA, and GC conceived and designed the experiments. SS and GC were responsible for the supervision of students and technicians. SS, KBH, US, and GD conducted molecular and alfalfa transformation experiments. SS, US, and KK carried out morphological and drought tolerance assessments. SS, KBH, and US wrote the manuscript. All authors were involved in revision of the manuscript.

## Conflict of Interest

The authors declare that the research was conducted in the absence of any commercial or financial relationships that could be construed as a potential conflict of interest.

## Publisher’s Note

All claims expressed in this article are solely those of the authors and do not necessarily represent those of their affiliated organizations, or those of the publisher, the editors and the reviewers. Any product that may be evaluated in this article, or claim that may be made by its manufacturer, is not guaranteed or endorsed by the publisher.

## Footnotes

1
www.alfalfatoolbox.org

2
http://plantgrn.noble.org/LegumeIP

3
http://plantgrn.noble.org/AGED/

4
http://smart.embl-heidelberg.de/smart/set_mode.cgi?NORMAL=1

5
https://play.google.com/store/apps/details?id=com.petioleapp.petiole&hl=en_CA&gl=US

6
https://petioleapp.com
