# Supplementary material for: The CRISPR/Cas9-Mediated Modulation of SQUAMOSA PROMOTER-BINDING PROTEIN-LIKE 8 in Alfalfa Leads to Distinct Phenotypic Outcomes
Source: Front Plant Sci. 2022 Jan 5;12:774146. doi: 10.3389/fpls.2021.774146 (PMC8793889; doi:10.3389/fpls.2021.774146)
Supplement: Supplementary file 1 [file Presentation_1.pptx]

## Slide 1
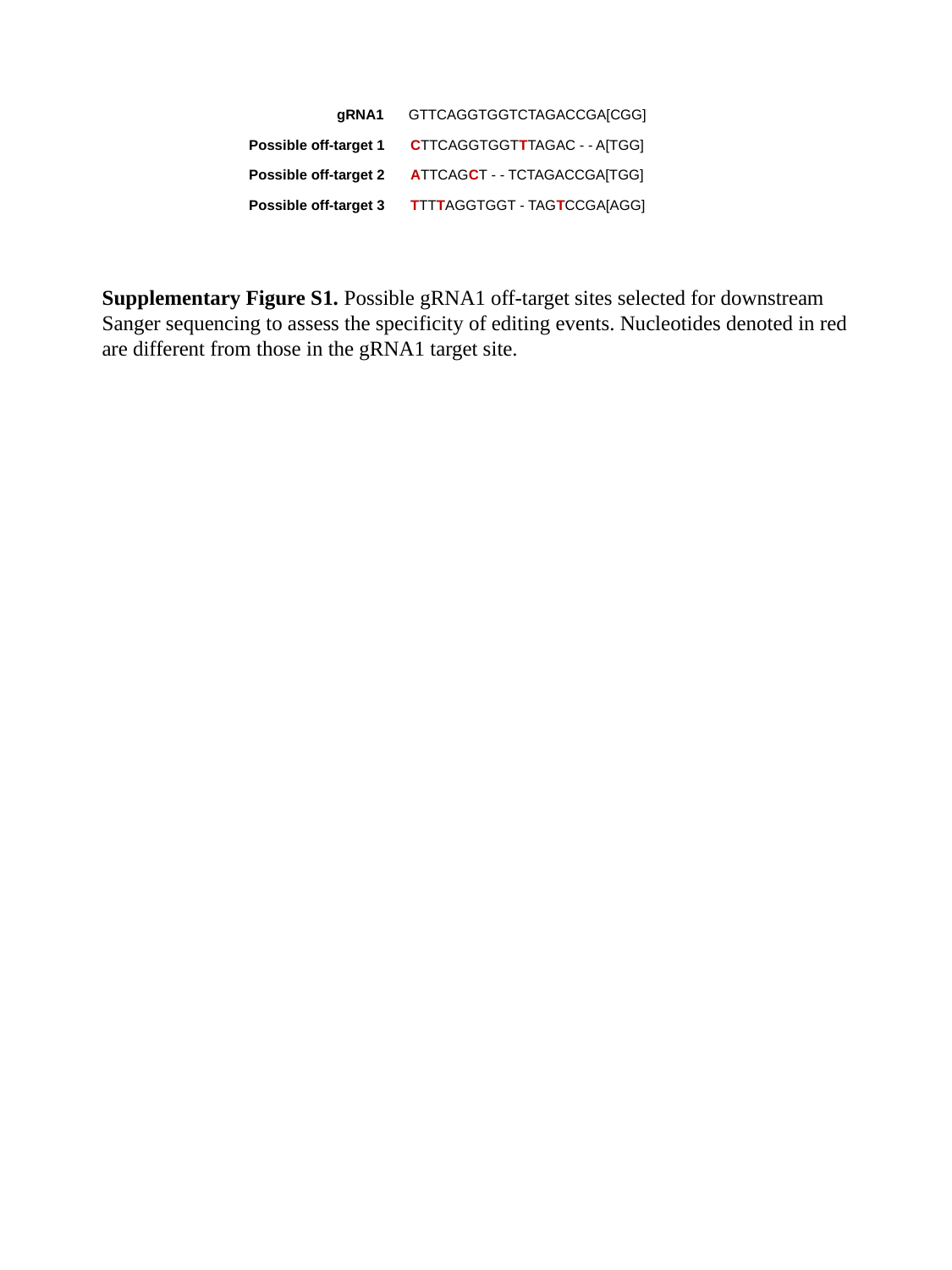

gRNA1
GTTCAGGTGGTCTAGACCGA[CGG]
Possible off-target 1
CTTCAGGTGGTTTAGAC - - A[TGG]
Possible off-target 2
ATTCAGCT - - TCTAGACCGA[TGG]
Possible off-target 3
TTTTAGGTGGT - TAGTCCGA[AGG]
Supplementary Figure S1. Possible gRNA1 off-target sites selected for downstream Sanger sequencing to assess the specificity of editing events. Nucleotides denoted in red are different from those in the gRNA1 target site.

## Slide 2
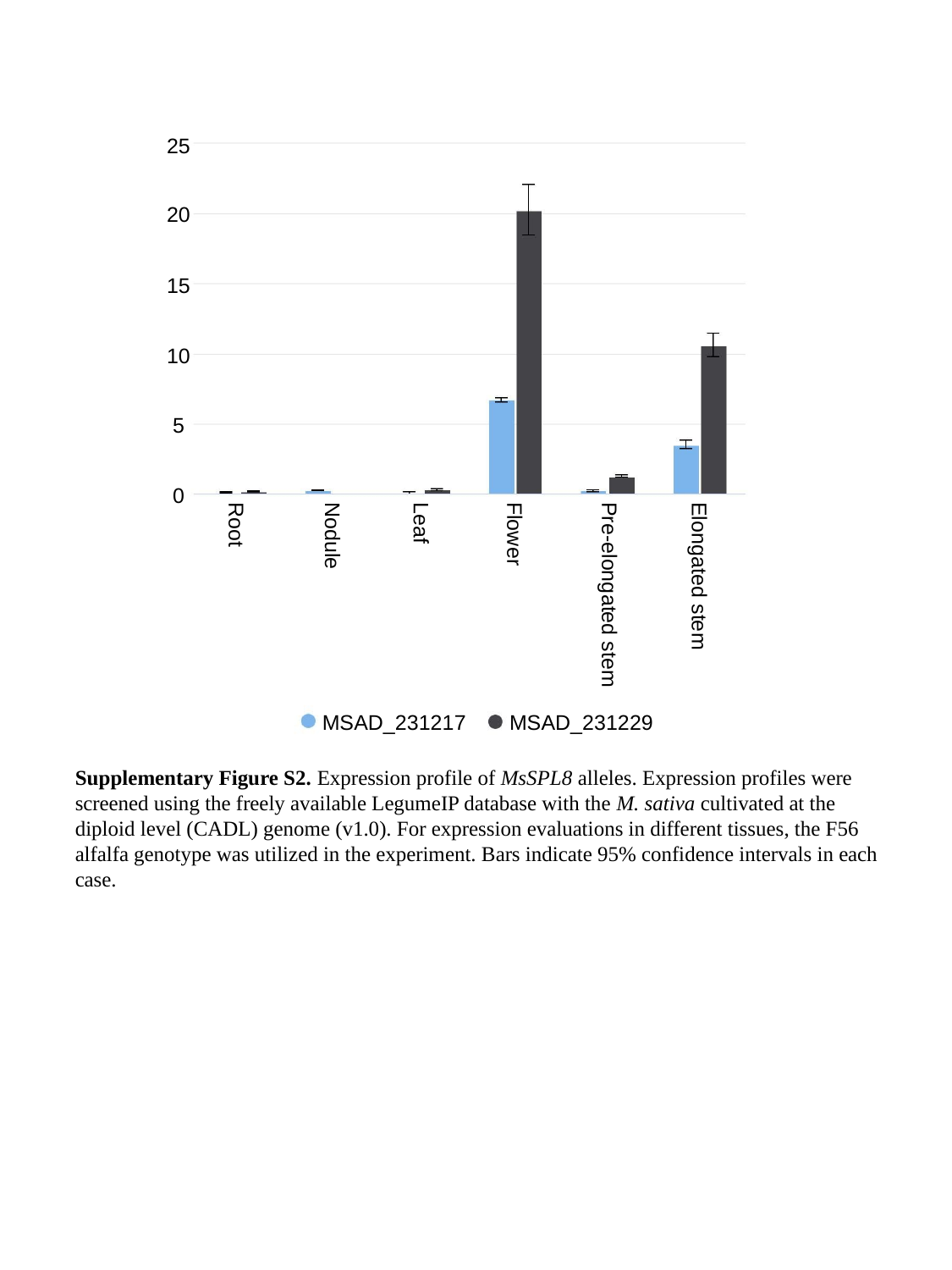

25
20
15
10
5
0
Root
Flower
Nodule
Leaf
Elongated stem
Pre-elongated stem
MSAD_231217
MSAD_231229
Supplementary Figure S2. Expression profile of MsSPL8 alleles. Expression profiles were screened using the freely available LegumeIP database with the M. sativa cultivated at the diploid level (CADL) genome (v1.0). For expression evaluations in different tissues, the F56 alfalfa genotype was utilized in the experiment. Bars indicate 95% confidence intervals in each case.

## Slide 3
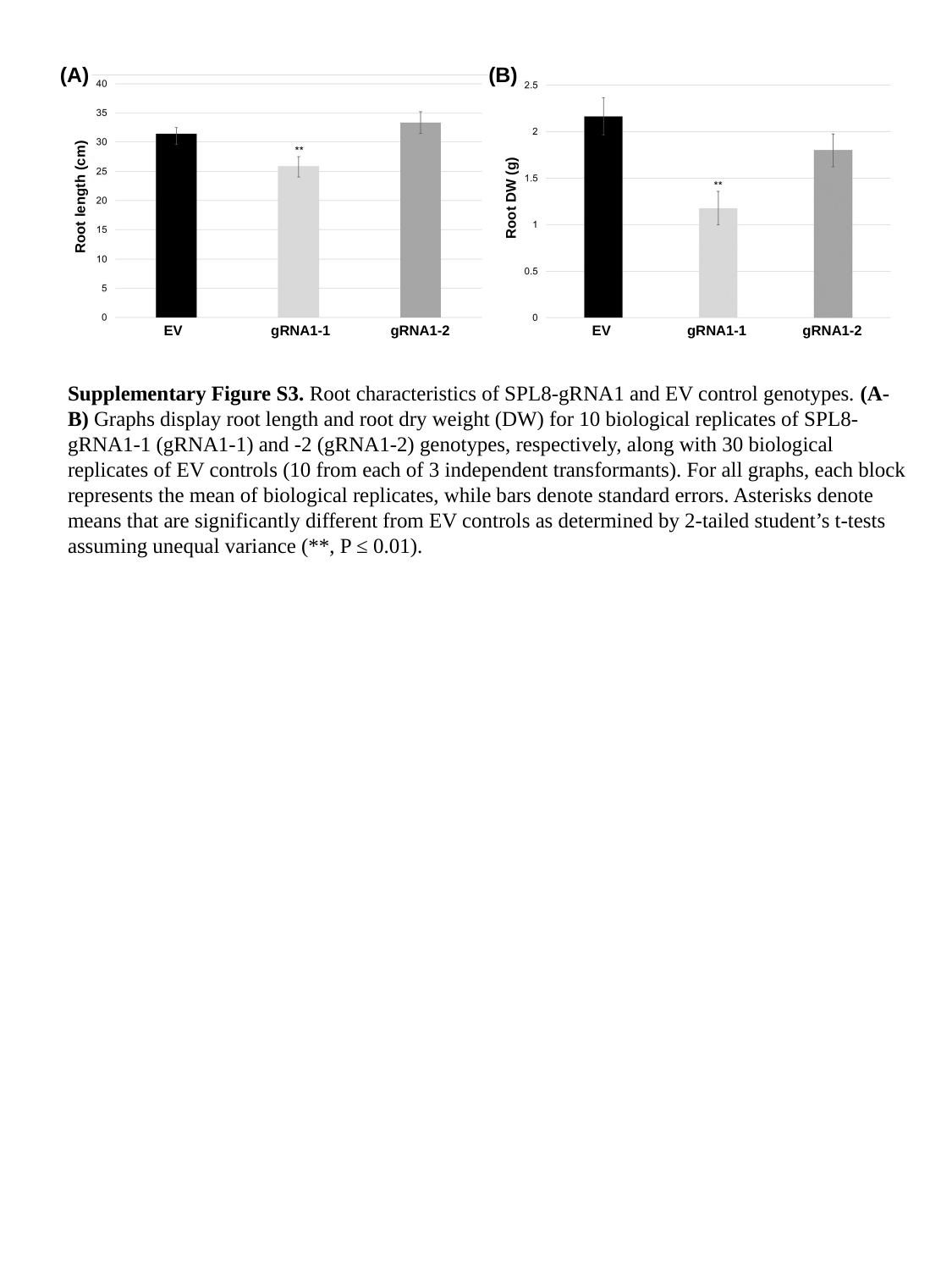

(A)
(B)
Root DW (g)
Root length (cm)
EV gRNA1-1 gRNA1-2
EV gRNA1-1 gRNA1-2
Supplementary Figure S3. Root characteristics of SPL8-gRNA1 and EV control genotypes. (A-B) Graphs display root length and root dry weight (DW) for 10 biological replicates of SPL8-gRNA1-1 (gRNA1-1) and -2 (gRNA1-2) genotypes, respectively, along with 30 biological replicates of EV controls (10 from each of 3 independent transformants). For all graphs, each block represents the mean of biological replicates, while bars denote standard errors. Asterisks denote means that are significantly different from EV controls as determined by 2-tailed student’s t-tests assuming unequal variance (**, P ≤ 0.01).

## Slide 4
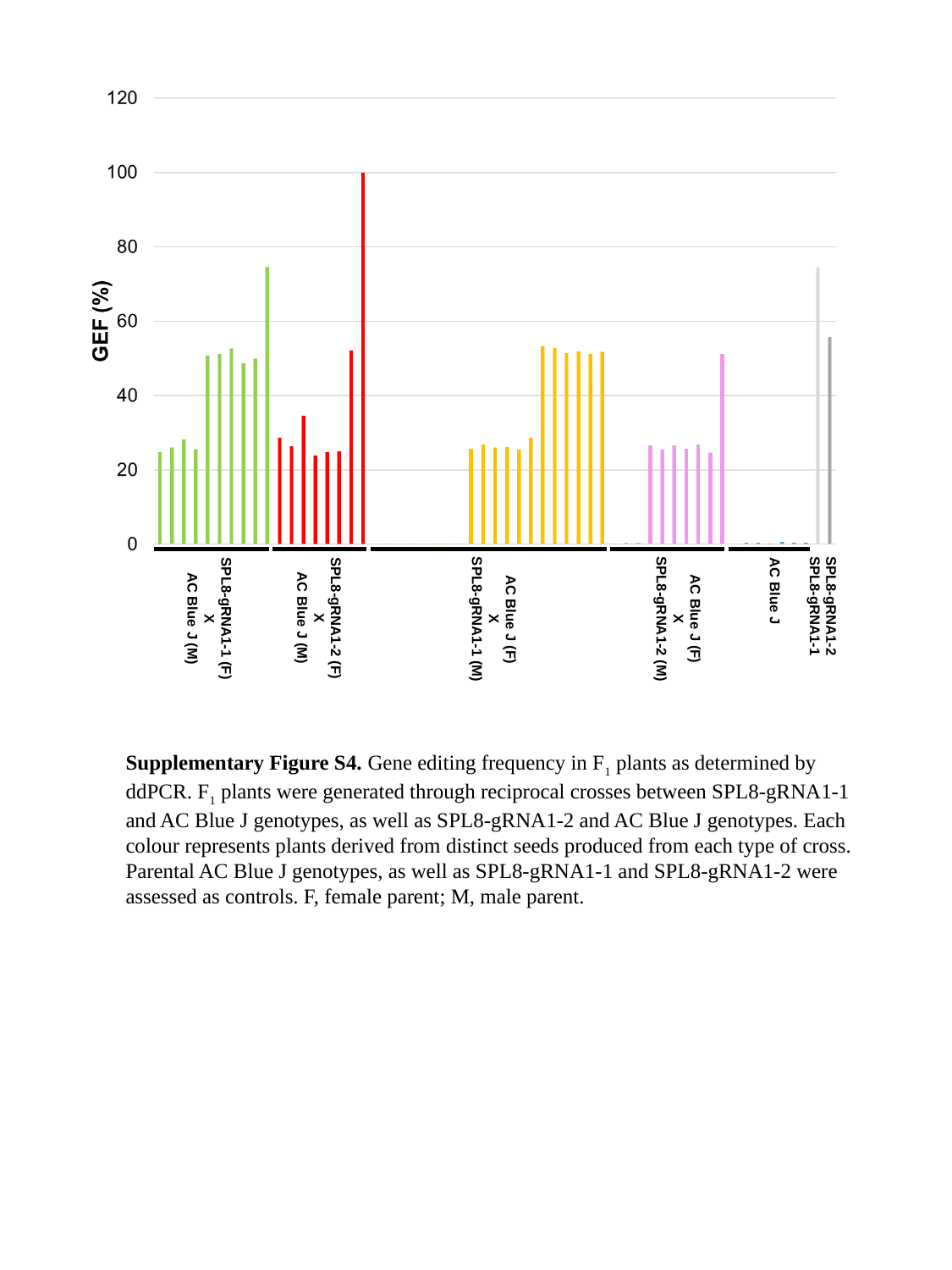

AC Blue J
SPL8-gRNA1-2 (F)
X
AC Blue J (M)
SPL8-gRNA1-1 (F)
X
AC Blue J (M)
AC Blue J (F)
X
SPL8-gRNA1-2 (M)
AC Blue J (F)
X
SPL8-gRNA1-1 (M)
SPL8-gRNA1-1
SPL8-gRNA1-2
Supplementary Figure S4. Gene editing frequency in F1 plants as determined by ddPCR. F1 plants were generated through reciprocal crosses between SPL8-gRNA1-1 and AC Blue J genotypes, as well as SPL8-gRNA1-2 and AC Blue J genotypes. Each colour represents plants derived from distinct seeds produced from each type of cross. Parental AC Blue J genotypes, as well as SPL8-gRNA1-1 and SPL8-gRNA1-2 were assessed as controls. F, female parent; M, male parent.
