# Supplementary material for: The CRISPR/Cas9-Mediated Modulation of SQUAMOSA PROMOTER-BINDING PROTEIN-LIKE 8 in Alfalfa Leads to Distinct Phenotypic Outcomes
Source: Front Plant Sci. 2022 Jan 5;12:774146. doi: 10.3389/fpls.2021.774146 (PMC8793889; doi:10.3389/fpls.2021.774146)
Supplement: Supplementary file 2 [file Data_Sheet_1.docx]

**Supplementary Table S1.** Primer sequences.

| **Primer** | **Sequence (5’ - 3’)** |
| --- | --- |
| MsSPL8Fwd2 | GCA CCA TAT CCA TCA GTC CTT C |
| MsSPL8Rev2 | TAG TGC TTC GCC TGA GAT AGA |
| MsSPL8Fwd3 | CTA TCT CAG GCG AAG CAC TAC |
| MsSPL8Rev3 | GAT CAC AAC CTG CTG CAT TG |
| MsSPL8F2 | TGG TCA TGG ACA TGG ACA TGG |
| MsSPL8F1 | TGG ACT ATG AAT GGG GTA ACC |
| MsSPL8R2 | CAA TCT CTT CCT GCA GCT ACG |
| MsOffTarget1FWD1  MsOffTarget1REV1 | CCT TTG ACC GCT TTC ACT CTA  GTG GGT CCA GTC TAT CCA TTT C |
| MsOffTarget2FWD1 | GGG TAC AAA GTA GTC GAT ACA TCA |
| MsOfTarget2REV1 | TTA TGG GAC AGA GGG AGT AGA G |
| MsOffTarget3FWD1 | CAG TTG CAT GGT TGG GAT ATT G |
| MsOffTarget3REV1 | CTC AAG TTC ATT TGG AGG CAT TT |
| Cas9F1 | GCT GGA GGA GTC ATT CCT CG |
| Cas9R1 | CTG AGA ATA TCA GAC AGG AGG |
| WD40F2 | GAA ACA AGA CTC GAG CAG TAC |
| WD40R2 | AAG CTC TCC TTG AAA ACC TCC |

**Supplementary Table S2.** GEF-ddPCR probe sequences.

| **gRNA** | **Type** | **Label** | **Sequence (5’ - 3’)** |
| --- | --- | --- | --- |
| gRNA1 | Edit | HEX | TA**G ACC** GA**C** GG |
| gRNA2 | Edit | HEX | TGA **GGT G**GA GG |
| gRNA3 | Edit | HEX | C**G**C **TGA G**T**C** AA |
| gRNA1/2 | Control | FAM | AAG AGA AGT ATG TGC GGC CAC CAA |
| gRNA3 | Control | FAM | TGG CTG CCT TGG AGT GAA ACT CA |

Note: Locked nucleic acid (LNA) bases are indicated in bold.
